# Research on Beef Marbling Grading Algorithm Based on Improved YOLOv8x

**DOI:** 10.3390/foods14101664

**Published:** 2025-05-08

**Authors:** Jun Liu, Lian Wang, Huafu Xu, Jie Pi, Daoying Wang

**Affiliations:** 1 College of Artificial Intelligence, Nanjing Agricultural University, Nanjing 210031, China; fakeforawhile@163.com; 2 Guangxi Key Laboratory of Digital Infrastructure, Guangxi Zhuang Autonomous Region Information Center, Nanning 530201, China; xuhf@gxi.gov.cn; 3 Institute of Agricultural Facilities and Equipment, Jiangsu Academy of Agricultural Sciences, Nanjing 210014, China; pijie@jaas.ac.cn; 4 Institute of Agricultural Products Processing, Jiangsu Academy of Agricultural Sciences, Nanjing 210014, China; daoyingwang@yahoo.com

**Keywords:** beef marbling, deep learning, YOLOv8x, ROI algorithm, machine vision, grading

## Abstract

Marbling is a crucial indicator that significantly impacts beef quality grading. Currently, Chinese beef processing enterprises rely on professional graders who visually assess marbling using national standard atlases. However, this manual evaluation method is highly subjective and time consuming. This study proposes a beef marbling grading algorithm based on an enhanced YOLOv8x model to address these challenges. The model integrates a convolutional neural network (CNN) augmented with an improved attention mechanism and loss function, along with a Region-of-Interest (ROI) preprocessing algorithm to automate the marbling grading process. A dataset comprising 1300 beef sample images was collected and split into training and test sets at an 8:2 ratio. Comparative experiments were conducted with other deep learning models as well as ablation tests to validate the proposed model’s effectiveness. The experimental results demonstrate that the improved YOLOv8x achieves a validation accuracy of 99.93%, a practical grading accuracy of 97.82%, and a detection time of less than 0.5 s per image. The proposed algorithm enhances grading efficiency and contributes to intelligent agricultural practices and livestock product quality assessment.

## 1. Introduction

Beef quality is graded based on the marbling, physiological maturity, muscle color, and fat color. Among these, the degree of marbling is the most important index to evaluate [1]. At present, the evaluation of beef marbling in China is mainly based on the national standard GB/T 29392-2022 [2]. The intramuscular fat distribution characteristics of the longissimus dorsi muscle between the 11th and 13th ribs were manually examined, and the marbling was scored through visual inspection by professional raters or using standard reference plates [3]. The United States Department of Agriculture (USDA) criteria emphasize fat distribution at the 12th rib, emphasizing beef maturity as much as beef marbling. In contrast, Australia emphasizes the uniformity of distribution of the beef fat particles [4]. However, no matter the kind, the manual discrimination method requires a lot of manpower, and due to human subjectivity, it is prone to inaccurate scoring, which is not suitable for automated production processes [5].

In recent years, numerous researchers, both domestically and internationally, have used machine vision technology to investigate and analyze beef marbling grading. L. Pannier et al. [6] conducted visual scanning and intramuscular fat (IMF%) testing on tenderloin samples, using the Australian beef marbling grading standard. Their findings revealed significant differences in marbling scores and IMF% across various sections (equivalent to 316 MSA units), indicating that single-site marbling measurements lead to more accurate steak grading. SM Stewart et al. [7] evaluated the predictive performance of chemical intramuscular fat (IMF%), MSA marbling score, and eye muscle area (EMA) using the Meat Imaging Japan (MIJ) prototype camera system. The results indicate that the prediction accuracy of the two cameras varies: IMF% (R^2^ = 0.4–0.5, RMSECV = 1.5–1.6%), MSA score (R^2^ = 0.3–0.5, RMSECV = 57.5–59.3), and EMA (R^2^ = 0.6–0.7, RMSECV = 4.1–5.8 cm^2^). Cai J et al. [8] combined broadband structural illumination reflection imaging, capturing images through sinusoidal illumination and decomposing them into DC and AC components. They employed machine learning models to achieve accurate segmentation and classification, with SegFormer performing the best, resulting in a classification accuracy of 90.85%. However, the recognition accuracy of the aforementioned machine learning algorithms is highly dependent on the effectiveness of feature extraction, and the accuracy of grading beef marbling requires further improvement.

With the progress of artificial intelligence technology and the improvement of computer hardware, deep learning has changed the traditional machine learning algorithm. Deep learning can automatically extract complex features from large datasets, reducing the dependence of traditional algorithms on manual feature engineering. A convolutional neural network (CNN) is a famous deep learning technology that has made a breakthrough in meat image processing and has been applied to food-related fields, such as detecting ham grade or defects, food quality, or microbial contamination. Munoz I et al. [9] proposed a method of using a CNN to segment the intramuscular fat of dried ham, which achieved a pixel accuracy of 0.99. The recall and precision of the segmented fat were close to 0.82 and 0.84, respectively. Gorji et al. [10] proposed a deep learning method combined with fluorescence imaging to detect microbial contamination areas on the surface of excised meat and achieved an accuracy of 97.32% in distinguishing clean and contaminated areas of the carcass. Cengel et al. [11] proposed a deep learning method to identify cracks and surface damage in eggs, and the recognition accuracy of damaged and undamaged eggs reached 98.73%.

YOLOv8 is the latest object detection model developed by Ultralytics (Frederick, MD, USA), providing improved accuracy and speed in detecting objects [12]. Unlike its predecessors, YOLOv8 features an optimized network architecture that supports various tasks, including object detection, image segmentation, and pose estimation, all while maintaining a lightweight design that ensures high performance [13]. The model also includes a modular design and utilizes mixed-precision training, which helps streamline both the training and deployment processes [14]. As a result, as an advanced computer vision algorithm, YOLOv8 has shown wide applicability in the field of agriculture and animal husbandry [15]. Its application scenarios include but are not limited to meat quality grading, carcass segmentation in slaughter automation, animal pathological tissue identification (such as parasite or inflammatory area detection) [16], and livestock and poultry health monitoring based on behavioral characteristics [17].

This paper introduces a novel deep learning model designed for beef marbling grading, aligning with industry requirements. The model is based on the YOLOv8x architecture and incorporates the convolutional block attention module (CBAM) along with Squeeze-and-Excitation (SE) attention mechanisms to enhance its feature extraction capabilities. Additionally, it employs Complete Intersection over Union (CIoU) as the loss function, which significantly improves the regression performance of the prediction box.

## 2. Materials and Methods

### 2.1. Dataset and Preprocessing

#### 2.1.1. Image Acquisition

**Figure 1:**
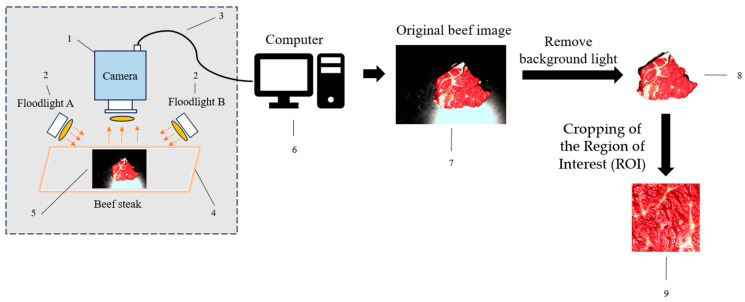
Schematic diagram of beef marbling image acquisition system. (1) Camera; (2) RGB fill light; (3) optical fiber cable; (4) sample placement table; (5) beef sample; (6) computer; (7) original beef image; (8) image of beef marbling after removing the background; (9) ROI image of beef marbling.

The sample used in this study is the longissimus dorsi muscle of beef (From Xiaolingwei Farmers Market, Nanjing, China), collected after cutting. The images were captured on the test bench at the Institute of Agricultural Facilities and Equipment, Jiangsu Academy of Agricultural Sciences. A schematic diagram of the image acquisition system used on the test bench is presented in Figure 1. The test bench was primarily composed of an RGB light source with a power controller (From Shenzhen Jinggou Technology Co., Ltd., Shenzhen, China), an industrial CCD camera (Hikvision MV-CE100-30GC) (From Hangzhou Hikvision Digital Technology Co., Ltd., Hangzhou, China) equipped with a zoom lens, and a desktop computer (From Beijing Legend Holdings Co., Ltd., Beijing, China). The image acquisition device had an effective resolution of 15 million pixels. To minimize ambient light interference during sample collection, indoor lights were turned off. The camera was positioned vertically, approximately 20 cm above the sample, while the light source was tilted about 12° relative to the vertical axis and positioned 15 cm away from the sample. The images were captured at a resolution of 2048 × 2048 pixels, and the original images were saved in .jpg format. A total of 1400 images were taken, from which 1300 high-quality beef image datasets were manually selected.

Firstly, the background of the original image was removed by the adaptive background light compensation algorithm, and then the beef marbling feature region was extracted based on the Region-of-Interest (ROI) algorithm. Batch image cropping technology was used to standardize the preprocessed ROI area, and the cropping size was 256 × 256 pixels. The experimental dataset was divided into a training set (80%) and a validation set (20%), where the training set contained 1040 samples and the validation set contained 260 samples. In the annotation process, the LabelImg tool was used to annotate the levels of the preprocessed images and generate an XML annotation file conforming to the PASCAL VOC format [18,19,20,21,22].

#### 2.1.2. Test Materials

In this study, the experimental base of Agricultural Facilities and Equipment Research Institute of Jiangsu Academy of Agricultural Sciences was selected as the experimental site, and the samples were obtained from the longissimus dorsi of cattle from three different farmers’ markets in Xuanwu District, Nanjing City, on 17 October 2024. The beef samples were preprocessed according to the following guidelines. Each muscle was cut to a standard thickness of 2.54 cm (1 inch) along the direction of the muscle fiber and left in a 4 °C incubator for 45 min to complete the color stabilization process (myoglobin oxidation was complete, and the surface color reached a bright-red steady state). According to the standard operating procedure of GB/T 29392-2022 “Quality Grading of livestock and poultry Meat Beef” [23], three specially trained personnels’ classifications under the standard light source were observed under light (2000 + 100 lux) for certain parts of a cattle carcass (usually 12–13 or 6–7 thoracic intercostal muscle cross-sectional longest back) with regard to intramuscular fat distribution, namely the marbled richness and evenness. The specific steps were as follows: select the observation area; take the cross-section of beef pectoralis dorsal longus muscle as the evaluation object; refer to the marbling grade map in the Chinese national standard; compare the size, density, and distribution uniformity of fat particles between the sample and the standard map by naked eyes. The personnel grading process integrates subjective evaluation experience with objective evidence analysis, followed by a final grade determination procedure. (For example, high grades require the fat particles to be dense and evenly distributed, while low grades require fat to be sparse).

**Figure 2:**
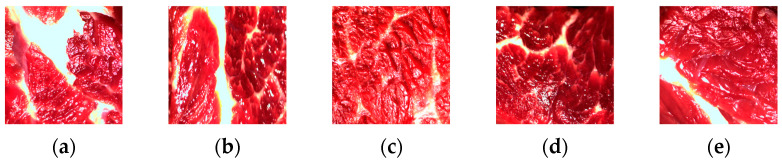
Beef original image dataset. (**a**) Prime. (**b**) Premium. (**c**) Second. (**d**) Third. (**e**) Fourth.

The experimental material contained 150 beef samples covering different degrees of marbling, divided into five categories according to grade: Prime-grade beef, Premium-grade beef, Second-grade beef, Third-grade beef, and Fourth-grade beef. Each category contained 30 samples, which were further processed and analyzed by image processing. Figure 2 shows an example of beef marbling in the original datasets.

### 2.2. YOLOv8x Network Model Improvement

#### 2.2.1. Embedding of CBAM Attention Mechanism Module

**Figure 3:**
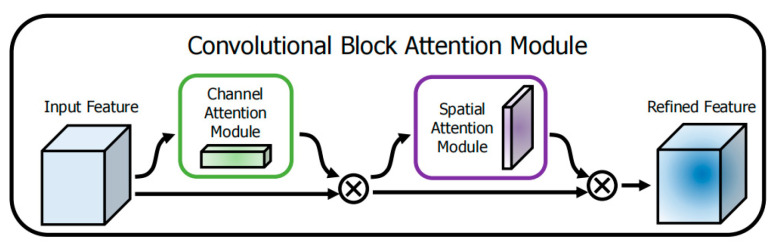
CBAM attention architecture diagram.

In response to the need for beef marbling detection, this study integrated the convolutional block attention module (CBAM) into the improved YOLOv8x model [24]. This mechanism simulates the characteristics of human visual attention and automatically focuses on the fat distribution characteristics of the beef back muscle area through global scanning. In the specific implementation, the channel attention module (CAM) analyzes the deep features of fat texture (such as fat density differences), and the spatial attention module (SAM) accurately locates the distribution pattern of fat particles [25]. As shown in Figure 3, the dual-path attention collaborative working mechanism enables the model to capture the “quality” and “position” information of fat features at the same time, significantly improving the accuracy of the quantitative analysis of marbling. This design effectively solves the problem of insufficient recognition of fat-heterogeneity areas by traditional algorithms.

**Figure 4:**
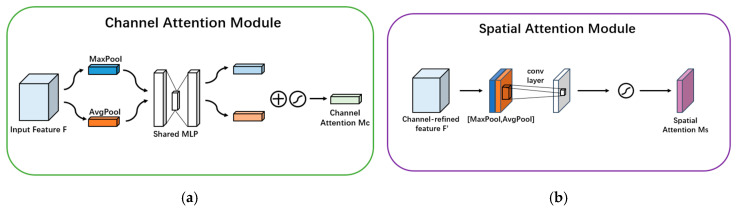
CAM and SAM attention mechanism flow chart. (**a**) CAM attention mechanism flow chart. (**b**) SAM attention mechanism flow chart.

The module has two sequential sub-modules: channel and spatial. The working principle of the channel attention module and the spatial attention module are shown in Figure 4.

#### 2.2.2. Embedding of SE Attention Mechanism Module

**Figure 5:**
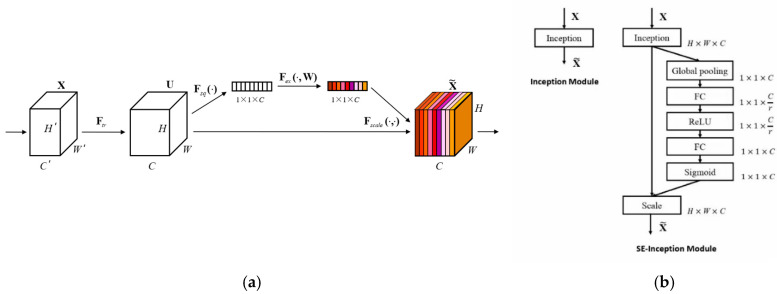
SE Attention mechanism calculation diagram. (**a**) Flow chart of SE processing. (**b**) Flow chart of the SE calculation.

This study uses the squeeze–excitation (SE) attention mechanism to dynamically strengthen the key feature channels of fat texture (such as the contrast between intramuscular fat and muscle bundles) by modeling inter-channel correlation and weakening redundant information such as the muscle background [26]. This mechanism adaptively adjusts the weights of each channel through the “squeeze-excitation” operation, allowing the model to focus on the core features of fat distribution in the beef muscle area, significantly improving the ability to distinguish fat–muscle texture in marbling grading [27]. The processing flow and calculation flow of the SE attention mechanism are shown in Figure 5.

#### 2.2.3. CIoU Loss Function Design

**Figure 6:**
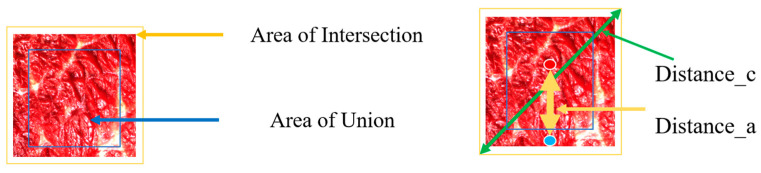
Comparison of loss function calculation frameworks.

Choosing an appropriate loss function can significantly improve the accuracy of beef-sized bounding box detection and ultimately achieve faster model convergence [28]. In the YOLOv8 model, the Intersection Over Union (IoU) loss function is used to evaluate the overlap degree between the predicted bounding box (predicted box) and the actual bounding box (real box) [29,30], while the CIoU loss function in this study can effectively distinguish the relative position relationship and reduce the object detection error [31]. The comparison of the computational flow framework between IoU and CIoU is shown in Figure 6.

**Figure 7:**
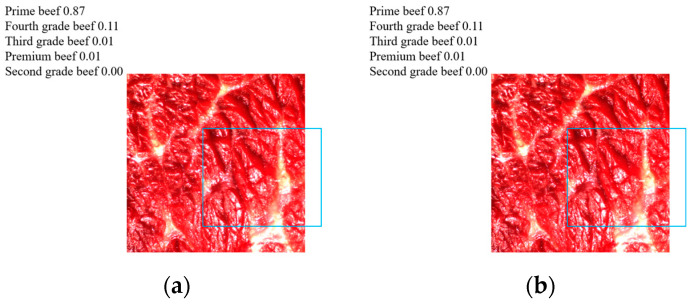
Box selection effects of different loss functions. (**a**) IoU detection effect diagram. (**b**) CIoU detection effect diagram.

This study used an improved CIoU loss function to optimize fat area positioning, as shown in Figure 7.

#### 2.2.4. Improved YOLOv8x Network Model

**Figure 8:**
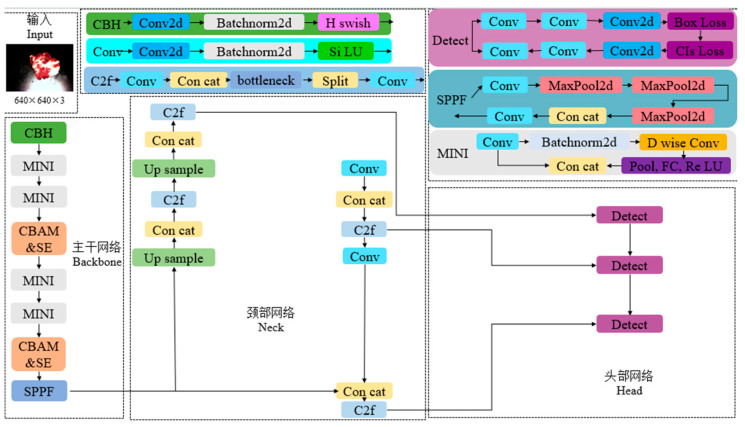
CBAM&SE-YOLOv8x-CIoU model architecture.

Aiming at the automatic grading task of beef marbling, this study significantly improved the grading accuracy of the model by improving the YOLO network. The dual attention mechanism of CBAM and SE was introduced to enable the model to dynamically focus on the key features of fat particle distribution in the rib eye region, which solves the problem of insufficient attention to local texture in traditional convolutional networks. At the same time, the loss function was improved to CIoU, and the Angle penalty mechanism was used to optimize the matching accuracy between the prediction box and the real annotation, which improves the accuracy of fat region localization. The whole improved model is shown in Figure 8.

#### 2.2.5. Evaluation Metrics

This study used a ratio of 8:2 to divide the training set and validation set and evaluate the accuracy of beef marbling recognition through four core indicators [32]. Precision: the proportion of pixels judged as “fat” by the model that are fat; accuracy: the overall recognition accuracy of fat and lean meat pixels (reducing misjudgment of lean meat); recall: the proportion of true fat pixels that are correctly identified (reducing missed fat detection); F1-Score: a balanced indicator of precision and recall [33]. TP: correctly identified fat (such as white fat patches in beef muscle); TN: correctly identified lean meat (red muscle area); FP: misjudged lean meat as fat (interfering texture); FN: misjudged fat as lean meat (missed small fat particles) [34]. This evaluation system can quantify the algorithm’s ability to capture the distribution of fat in beef marbling. The calculation formulas for Precision, Accuracy, Recall, and F1-Score value are as follows:

(1) $$Precision=\frac{T_{P}}{T_{P}+F_{P}},$$

(2) $$Accuracy=\frac{T_{P}+T_{N}}{T_{P}+T_{N}+F_{P}+F_{N}},$$

(3) $$Recall=\frac{T_{P}}{T_{P}+F_{N}},$$

(4) $$F1‐Score=\frac{2Precision\cdot Recall}{Precision+Recall},$$

Precision measures how accurately fat pixels are predicted, while accuracy reflects the overall correctness of all the pixel predictions [35]. Recall quantifies the proportion of actual fat pixels that were correctly predicted. The F1-Score, an important evaluation metric for deep learning models, combines precision and recall to provide a comprehensive assessment of the model’s prediction capabilities [36].

## 3. Results and Discussion

### 3.1. Experimental Platform and Model Training Results

The experimental training sets comprised 1040 beef sample images, while the validation sets contained 260 beef images. The deep learning framework used for this project was PyTorch 2.4.1 [33,34]. The deep learning processor utilized was the 13th Gen Intel(R) Core(TM) i9-13900HX at 2.20 GHz, paired with an RTX 4060 Laptop graphics card. The operating system was Windows 11, along with the NVIDIA 560.94 driver, CUDA version 12.6, and the cuDNN neural network acceleration library version 8.9.7.

Considering the hardware performance of the experimental platform, the batch size was set to 16, and the number of iterations was selected as 100 based on the model’s fitting results. To enhance model stability and prevent oscillation during the later stages of training, the momentum factor was set to 0.973, the decay coefficient to 0.005, and the initial learning rate to 0.001. The optimizer chosen was SGD, and both the Mosaic data enhancement algorithm and the ROI image processing algorithm were employed.

**Figure 9:**
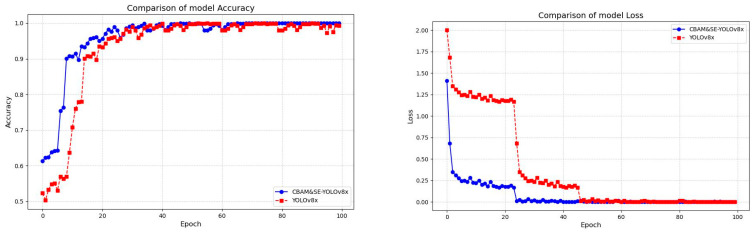
Comparison of model training performance.

The differences in training performance between the CBAM&SE-YOLOv8x and YOLOv8x models on the beef marbling dataset were compared, including the training effect and accuracy of recording the loss function value. The experiments showed that the accuracy of the improved model reached 99.4% in 35 epochs, which is 20.9% higher than that of the original model. The loss function value was stable at 0.008, a decrease of 98.4%, and it was 25 epochs earlier than the convergence round of the original model. This indicates that the training performance of the improved model is significantly better than that of the original model. The comparison chart of the training accuracy and loss values before and after the improvement of the model is shown in Figure 9.

**Figure 10:**
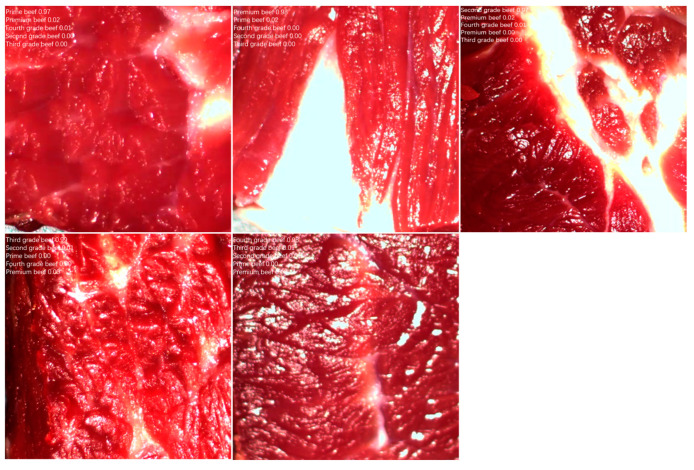
Grading results of beef marbling.

The final classification result of the beef marbling grade is shown in Figure 10. As shown in the figure, for the classification of five different beef marbling grades, such as super grade, excellent grade, second grade, third grade, and fourth grade, the final actual classification accuracy of the model reached more than 97%; especially, the actual classification accuracy of secondary beef was as high as 99%.

### 3.2. Comparative Experiments of Different Deep Learning Models

**Figure 11:**
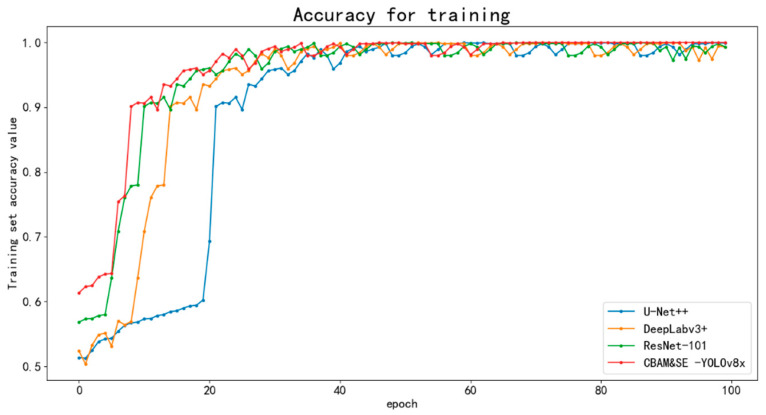
Accuracy iteration curve of the model.

This research aimed to improve the automation of beef quality evaluation and grading using advanced computer vision techniques. The goal was to significantly improve the speed and accuracy of the grading process. To comprehensively evaluate the performance of the improved model, several comparative experiments of deep learning models were designed and carried out. This experiment systematically evaluated the performance differences of different models in the beef marbling grading task. The evaluation models included the following: (1) U-Net++: a convolutional neural network with a nested skip connection architecture, which enhances the pixel-level segmentation ability of intermuscular adipose tissue through dense convolution blocks, and a multi-scale feature fusion mechanism that can accurately identify complex image texture boundaries [37]; (2) DeepLabv3+: introduces a spatial pyramid pooling module, which can effectively capture the information distribution in different regions of the image by using dilated convolution [38]; (3) ResNet-101: based on the residual grouped convolution structure, the interaction efficiency between feature channels is improved through the optimization of cardinality parameters, and the robust efficiency of image deep feature extraction improved; (4) an optimized version of the YOLOv8x model, which was further improved in this study [39]. The accuracy training curves of the four deep learning models are compared in Figure 11. In the early stage of training, the U-Net++, DeepLabv3+, and ResNet-101 models have low recognition accuracy. In contrast, the CBAM&SE-YOLOv8x model has high initial accuracy. The training accuracy of the CBAM&SE-YOLOv8x model tends to be stable after the 20th training cycle, and the performance is close to the optimal after the 60th cycle. Although the other models have low initial accuracy, they also successively reach the maximum value after the 80th cycle. The analysis results show that the CBAM&SE-YOLOv8x model performed best in the beef marbling classification recognition task, and the final training accuracy reached 99.99%.

This study applied the above model to the task of beef marbling grading and conducted comparative experiments with the optimized YOLOv8x model (CBAM&SE-YOLOv8x) to comprehensively evaluate the performance of each model in terms of grading Accuracy, Mean average recognition accuracy (mAP), Recall, F1-Score. The experimental results show that the proposed optimized model CBAM&SE-YOLOv8x was superior to the other comparison models in all indicators. Table 1 shows the comparison results of each model on the test set in detail, including key evaluation indicators such as Accuracy, mAP, Recall, F1, and single-image-detection processing speed.

### 3.3. YOLOv8x Model Ablation Test

To verify the embedding effect of the CBAM and SE attention mechanism and the improved performance of the CIoU loss function design, this study designed and implemented ablation experiments using mAP, number of parameters, and model weight as the performance evaluation indicators. The experimental results are shown in Table 2.

As shown in Table 2, the mAP of the model increased by 0.9 percentage points after adding the CBAM attention mechanism, increased by 2.7 percentage points after adding the CBAM attention mechanism and SE attention mechanism, and increased by 7.2 percentage points after continuing to embed the CIoU loss function. The number of parameters and the weights of the model were significantly reduced after the improvement.

### 3.4. Comparison of Image Acquisition Methods

Compared with the Q-FOM camera used by Stewart et al., the Q-FOM camera system assesses chemical fat (IMF%) in muscle and predicts its precision and accuracy based on the Meat Standards Institute of Australia (MSA) marbling score and Meat Eye Muscle Area of Australia (EMA). The image acquisition system used in this study is based on the national standard GB/T 29392-2022 “Quality Classification of Livestock and Poultry Meat and Beef” to evaluate the precipitation richness of beef fat. The quality grade of beef was predicted based on the fat richness. These standards have different detection emphases, but both of them can achieve the purpose of grading beef marbling.

## 4. Conclusions

The beef industry is one of the most important meat industries in our country. Nondestructive testing of beef quality is of great significance to promoting the sustainable development of the economy and society in China and ensuring food safety. In this study, an improved CBAM&SE-YOLOv8x model is proposed to improve the accuracy and efficiency of beef marbling detection. As the core link of food quality testing, target detection technology has important application value in ensuring the standardization of meat grading. The model takes YOLOv8x as the benchmark architecture, innovatively integrates the CBAM attention mechanism and SE attention mechanism into the backbone network, enhances the relationship modeling between feature channels and the important information focus of spatial dimensions and significantly improves the extraction ability of key features. At the same time, the original IoU loss function is replaced by the CIoU loss function considering the parameters of overlap area, center point distance, and aspect ratio. This improvement not only optimizes the accuracy of object box regression but also effectively alleviates the gradient disappearance problem of traditional IoU.

Experiments on the beef marbling training dataset show that the improved model showed significant advantages. Compared with the original model, the parameter compression rate was 79.9%; the Mean Average Precision (mAP) increased by 7.2 percentage points; and the processing time of a single frame was shortened by 30.1 ms. It is particularly noteworthy that the optimized model only needed 41.28 MB of memory to achieve a real-time detection speed of 5.3 ms, which ensures detection efficiency while remaining lightweight. The focusing ability of texture features improved by 23%, and the positioning error of small target detection reduced by 18.6%.

In addition, given the complex texture features of beef, future research can start with multi-modal data fusion to fuse multi-source information such as NIR spectroscopy and texture images. Unsupervised domain adaptation methods can also be explored in the future to alleviate the impact of labeled data scarcity on model generalization ability. In terms of model architecture, future attempts can be made to combine the causal inference framework with the attention mechanism to construct interpretable causal graphs to guide feature selection. Developing a model deployment scheme for edge computing devices and focusing on the application of deep learning models in nondestructive testing algorithms in real industries to provide more accurate and deeper insights for beef quality prediction should be the goal of future research.

## Figures and Tables

**Table 1:** Comparative experiment of deep learning models.

Model	Accuracy (%)	mAP (%)	Recall (%)	F1(%)	Rate (ms/sheet)
U-Net++	99.48	99.36	93.26	85.96	35.4
DeepLabv3+	96.8	96.53	96.32	86.72	37.5
ResNet-101	97.62	97.53	95.78	88.72	28.2
CBAM&SE-YOLOv8x	99.99	99.99	99.49	99.53	5.3

**Table 2:** Ablation test.

CBAM	SE-Attention	CIoU	mAP (%)	Params (M)	Weights (MB)
-	-	-	92.7	61.98	232.82
√	-	-	93.6	12.52	44.56
√	√	-	95.4	12.48	41.28
√	√	√	99.9	12.48	41.28

Note: “√” means adding the module; “-” means not adding the module.

## Data Availability

No new data were created or analyzed in this study. Data sharing is not applicable to this article.

## References

[1] Smith S.B., Gotoh T., Greenwood P.L. (2018). Current Situation and Future Prospects for Global Beef Production: Overview of Special Issue. Asian-Australas. J. Anim. Sci..

[2] Li X.Z., Yan C.G., Zan L.S. (2018). Current Situation and Future Prospects for Beef Production in China—A Review. Asian-Australas. J. Anim. Sci..

[3] Cheng W., Cheng J., Sun D., Pu H. (2015). Marbling Analysis for Evaluating Meat Quality: Methods and Techniques. Comp. Rev. Food Sci. Food Safe.

[4] Clinquart A., Ellies-Oury M.P., Hocquette J.F., Guillier L., Santé-Lhoutellier V., Prache S. (2022). Review: On-Farm and Processing Factors Affecting Bovine Carcass and Meat Quality. Animal.

[5] Huerta-Leidenz N. (2021). Progress on Nutrient Composition, Meat Standardization, Grading, Processing, and Safety for Different Types of Meat Sources. Foods.

[6] Pannier L., Van De Weijer T.M., Van Der Steen F.T.H.J., Kranenbarg R., Gardner G.E. (2023). Adding Value to Beef Portion Steaks through Measuring Individual Marbling. Meat Sci..

[7] Stewart S.M., Gardner G.E., Williams A., Pethick D.W., McGilchrist P., Kuchida K. (2021). Association between Visual Marbling Score and Chemical Intramuscular Fat with Camera Marbling Percentage in Australian Beef Carcasses. Meat Sci..

[8] Cai J., Lu Y., Olaniyi E., Wang S., Dahlgren C., Devost-Burnett D., Dinh T. (2024). Beef Marbling Assessment by Structured-Illumination Reflectance Imaging with Deep Learning. J. Food Eng..

[9] Muñoz I., Gou P., Fulladosa E. (2019). Computer Image Analysis for Intramuscular Fat Segmentation in Dry-Cured Ham Slices Using Convolutional Neural Networks. Food Control.

[10] Gorji H.T., Shahabi S.M., Sharma A., Tande L.Q., Husarik K., Qin J., Chan D.E., Baek I., Kim M.S., MacKinnon N. (2022). Combining Deep Learning and Fluorescence Imaging to Automatically Identify Fecal Contamination on Meat Carcasses. Sci. Rep..

[11] Cengel T.A., Gencturk B., Yasin E.T., Yildiz M.B., Cinar I., Koklu M. (2025). Automating Egg Damage Detection for Improved Quality Control in the Food Industry Using Deep Learning. J. Food Sci..

[12] Hussain M. (2023). YOLO-v1 to YOLO-v8, the Rise of YOLO and Its Complementary Nature toward Digital Manufacturing and Industrial Defect Detection. Machines.

[13] Lou H., Duan X., Guo J., Liu H., Gu J., Bi L., Chen H. (2023). DC-YOLOv8: Small-Size Object Detection Algorithm Based on Camera Sensor. Electronics.

[14] Ye S., Zhang S., Meng Q., Wang H., Zhu J. Disease Detection Module for SBCE Images Using Modified YOLOv8. Proceedings of the 2024 IEEE International Conference on Systems, Man, and Cybernetics (SMC).

[15] Hussain M. (2024). YOLOv1 to v8: Unveiling Each Variant–A Comprehensive Review of YOLO. IEEE Access.

[16] Badgujar C.M., Poulose A., Gan H. (2024). Agricultural Object Detection with You Look Only Once (YOLO) Algorithm: A Bibliometric and Systematic Literature Review. Comput. Electron. Agric..

[17] Wu X., Tang R., Mu J., Niu Y., Xu Z., Chen Z. (2024). A Lightweight Grape Detection Model in Natural Environments Based on an Enhanced YOLOv8 Framework. Front. Plant Sci..

[18] Farahmand S., Fernandez A.I., Ahmed F.S., Rimm D.L., Chuang J.H., Reisenbichler E., Zarringhalam K. (2022). Deep Learning Trained on Hematoxylin and Eosin Tumor Region of Interest Predicts HER2 Status and Trastuzumab Treatment Response in HER2+ Breast Cancer. Mod. Pathol..

[19] Oyelade O.N., Ezugwu A.E., Almutairi M.S., Saha A.K., Abualigah L., Chiroma H. (2022). A Generative Adversarial Network for Synthetization of Regions of Interest Based on Digital Mammograms. Sci. Rep..

[20] Dang M., Wang H., Li Y., Nguyen T.-H., Tightiz L., Xuan-Mung N., Nguyen T.N. (2024). Computer Vision for Plant Disease Recognition: A Comprehensive Review. Bot. Rev..

[21] Shi Y., Wang X., Borhan M.S., Young J., Newman D., Berg E., Sun X. (2021). A Review on Meat Quality Evaluation Methods Based on Non-Destructive Computer Vision and Artificial Intelligence Technologies. Food Sci. Anim. Resour..

[22] Zhang C., Zhang D., Su Y., Zheng X., Li S., Chen L. (2022). Research on the Authenticity of Mutton Based on Machine Vision Technology. Foods.

[23] Gao Q., Liu H., Wang Z., Lan X., An J., Shen W., Wan F. (2023). Recent Advances in Feed and Nutrition of Beef Cattle in China—A Review. Anim. Biosci..

[24] Zakariah M., Alnuaim A. (2024). Recognizing Human Activities with the Use of Convolutional Block Attention Module. Egypt. Inform. J..

[25] Wang S., Huang L., Jiang D., Sun Y., Jiang G., Li J., Zou C., Fan H., Xie Y., Xiong H. (2022). Improved Multi-Stream Convolutional Block Attention Module for sEMG-Based Gesture Recognition. Front. Bioeng. Biotechnol..

[26] Qin X., Li M., Liu Y., Zheng H., Chen J., Zhang M. (2022). An Efficient Coding-based Grayscale Image Automatic Colorization Method Combined with Attention Mechanism. IET Image Process..

[27] Zhou T., Ye X., Lu H., Zheng X., Qiu S., Liu Y. (2022). Dense Convolutional Network and Its Application in Medical Image Analysis. BioMed Res. Int..

[28] Wang Z., Sun W., Zhu Q., Shi P. (2022). Face Mask-Wearing Detection Model Based on Loss Function and Attention Mechanism. Comput. Intell. Neurosci..

[29] Goceri E. (2024). GAN Based Augmentation Using a Hybrid Loss Function for Dermoscopy Images. Artif. Intell. Rev..

[30] Zhang Y.-F., Ren W., Zhang Z., Jia Z., Wang L., Tan T. (2022). Focal and Efficient IOU Loss for Accurate Bounding Box Regression. Neurocomputing.

[31] Gao J., Chen Y., Wei Y., Li J. (2021). Detection of Specific Building in Remote Sensing Images Using a Novel YOLO-S-CIOU Model. Case: Gas Station Identification. Sensors.

[32] Hicks S.A., Strümke I., Thambawita V., Hammou M., Riegler M.A., Halvorsen P., Parasa S. (2022). On Evaluation Metrics for Medical Applications of Artificial Intelligence. Sci. Rep..

[33] Vujovic Ž.Ð. (2021). Classification Model Evaluation Metrics. IJACSA.

[34] Zhou J., Gandomi A.H., Chen F., Holzinger A. (2021). Evaluating the Quality of Machine Learning Explanations: A Survey on Methods and Metrics. Electronics.

[35] Paszke A., Gross S., Massa F., Lerer A., Bradbury J., Chanan G., Killeen T., Lin Z., Gimelshein N., Antiga L. PyTorch: An Imperative Style, High-Performance Deep Learning Library. Proceedings of the 33rd International Conference on Neural Information Processing Systems.

[36] Bartz-Beielstein T. (2023). PyTorch Hyperparameter Tuning—A Tutorial for spotPython 2023. arXiv.

[37] Wisaeng K. (2023). U-Net++DSM: Improved U-Net++ for Brain Tumor Segmentation With Deep Supervision Mechanism. IEEE Access.

[38] Chang Z., Li H., Chen D., Liu Y., Zou C., Chen J., Han W., Liu S., Zhang N. (2023). Crop Type Identification Using High-Resolution Remote Sensing Images Based on an Improved DeepLabV3+ Network. Remote Sens..

[39] Mahapatra S.K., Pattanayak B.K., Pati B. (2023). Attendance Monitoring of Masked Faces Using ResNext-101. JSMS.

